# A comparative analysis of GPT-3.5 and GPT-4.0 on a multiple-choice ophthalmology question bank: A study on artificial intelligence developments

**DOI:** 10.22336/rjo.2024.67

**Published:** 2024

**Authors:** Suleyman Demir

**Affiliations:** Department of Opthalmology, Adana 5 Ocak State Hospital, Adana, Turkey

**Keywords:** ChatGPT-3.5, ChatGPT-4.0, artificial intelligence, medical education, Ophthalmology, AI = Artificial intelligence, GPT = Generative Pretrained Transformer, LLMs = Large language models, OKAP = Ophthalmic Knowledge Assessment Programme, MCQs = Multiple-choice questions, USMLE = United States Medical Licensing Examination

## Abstract

**Introduction:**

To evaluate the performance of ChatGPT-4.0 and ChatGPT-3.5 in answering multiple-choice questions in OphthoQuestions (www.ophthoquestions.com), a popular question preparation bank, and to compare the performance of GPT-4.0 and GPT-3.5.

**Methods:**

In January 2024, using a personal account on OphthoQuestions (www.ophthoquestions.com), 520 questions were selected from 4,551 OphthoQuestions. These 520 questions were created by randomly selecting 40 questions from each of 13 ophthalmology subspecialties. GPT-3.5 and GPT-4.0 were asked to answer these same 520 questions.

**Results:**

ChatGPT-4.0 and ChatGPT-3.5 answered 408 questions (78.46%) 95% CI [70,88%] and 333 questions (64.15%) 95% CI [53,74%] of 520 questions correctly, respectively. GPT-4.0 answered significantly more questions correctly than GPT-3.5 (p= 0.0195). ChatGPT-4.0 showed a statistically significant difference compared to ChatGPT-3.5 in giving correct answers in all subgroup analyses (p<0.05).

**Discussions:**

This study gives an encouraging new proof of ChatGPT’s ability to manage complex clinical and medical data, focusing on the development and consistency of artificial intelligence algorithms. The statistically significant success of GPT-4.0 over GPT-3.5 in this study should be examined in light of future algorithm advances, particularly in online tests, which will increase progressively as the use of artificial intelligence poses an increasing danger to test integrity. Protocols such as required proctoring should be considered. In the following years, ChatGPT’s clinical management and decision-making expertise should be supplemented by more research indicating that it may be a beneficial resource for ophthalmologists and other medical professionals seeking information and guidance on challenging cases.

**Conclusions:**

GPT-4.0 was found to give more and more consistent answers than GPT 3.5 on a multiple-choice ophthalmology question bank. ChatGPT has shown significant differences between algorithms in accuracy and repeatability when handling questions related to eye diseases. This study shows that new artificial intelligence algorithms are promising. More data is needed to use artificial intelligence language models in medical applications.

## Introduction

The medical industry is among the many fields where artificial intelligence (AI) has shown increasing promise. In recent years, doctors have frequently used artificial intelligence to assist them in diagnosis, treatment, and research [1,2]. In the past, AI has been utilized to identify different retinal pathologies, such as age-related macular degeneration and diabetic retinopathy [3-5]. The literature also shows how AI can be helpful in conditions other than retinal pathologies [6].

Large language model (LLM) Generative Pretrained Transformer 3 (GPT-3) produces text that appears human. It received training on a vast corpus of text (more than 400 billion words) from the internet, which included webpages, books, and articles [7]. The large language model (LLM) ChatGPT (OpenAI, San Francisco, CA, USA) has caused a paradigm shift in the application of artificial intelligence in medicine [8,9]. Currently limited to training using online resources until September 2021, GPT-3.5 is an improved version of GPT-3 (2020) trained on a wide range of parameters [10,11]. In March 2023, OpenAI unveiled GPT-4, a new generation LLM that outperforms GPT-3.5 and performs at a human level across various academic benchmarks [12].

The large language models (LLMs) and text-based LLMs can potentially improve medical diagnosis and interpretation. OphthoQuestions question banks, the Basic and Clinical Sciences Course (BCSC) Self-Assessment Programme, and FRCOphth examinations have previously been used to test the effectiveness of these models, particularly in ophthalmology [13,14]. The performance of LLMs in ophthalmology question answering is still not sufficiently analyzed, although there are studies on their performance [15,16].

This study evaluated a comparative analysis of GPT-3.5 and GPT-4.0 on the multiple-choice ophthalmology question bank using OphthoQuestions (www.ophthoquestions.com), a popular question preparation bank. Ophthalmologists frequently consult this multiple-choice question bank as these resources have been linked to improved performance on the standardized Ophthalmic Knowledge Assessment Programme (OKAP) examination taken by ophthalmology residents in the United States and Canada, particularly in studying for board examinations.

## Methods

### Exploring OphthoQuestions

In January 2024, using a personal account on OphthoQuestions (www.ophthoquestions.com), 520 questions were selected from 4,551 OphthoQuestions. Since the GPT-3.5 and GPT-4.0 multiple-choice question bank performances were compared, using questions that did not contain visual data, such as clinical, radiological, or graphic images, was preferred since the GPT-3.5 model could not analyze visual data. These questions were not available to the general public, meaning there was no chance that they were previously indexed in the ChatGPT training data set or any search engine. The researcher generated 40 random questions from each of the 13 ophthalmology sub-specialties. These subgroups included general medicine, fundamentals, clinical optics, cornea, uveitis, glaucoma, lens and cataract, pathology and tumors, neuro-ophthalmology, pediatrics, oculoplastics, retina, vitreous, and refractive surgery.

### Study Design

The researcher manually entered the content of the text-based questions into the program. A new chat was opened for each question. Then, the statement “You should choose one of the following options” was written. Questions containing visual elements such as clinical images or medical photographs were not included in our evaluation as ChatGPT-3.5 could not analyze them. This study assessed the gross accuracy in correctly completing a series of multiple-choice questions (MCQs).

ChatGPT was considered to have given a “correct” answer for scoring purposes when it selected the option suggested by the answer key for a given question. On the other hand, an answer was considered “incorrect” if it did not match the answer's essential suggestion, if the platform failed to identify any option when asked further, or if the third attempt was incorrect in the case of conflicting duplicate answers. The answers were then checked against the answer key by the researcher, and the correct answers were analyzed according to subgroups and overall groups. A conservative analysis strategy was adopted, preferring not to set thresholds similar to those in other studies. Instead, the assessment of whether the performance of GPT-4.0 was different from GPT-3.5 was performed [14].

### Statistical analysis

To analyze categoric variables, Fisher’s exact test and Chi-square (X^2^) were used to compare the number of correct responses on the GPT-4.0 and GPT-3.5 tests. The Kolmogorov-Smirnov test was used to assess the data’s normality. The accuracy and compliance rates were reported in percentage numbers. The accuracy of the thirteen distinct subspecialties was also compared using chi-square analysis. A P-value of 0.05 was regarded as statistically significant. The studies used SPSS, version 25.0 (SPSS Inc., Chicago, IL, USA).

## Results

In general, GPT-4.0 and GPT-3.5 answered 408 questions (78.46%) 95% CI [70,88%] and 333 questions (64.15%) 95% CI [53,74%] of 520 questions correctly, respectively. GPT-4.0 answered statistically significantly more questions correctly compared to GPT-3.5 (p = 0.0195). Chat GPT 4.0 showed a statistically significant difference compared to Chat GPT 3.5 in giving correct answers in all subgroup analyses (p<0.05). In subgroup analyses, pathology and tumors were the groups with the highest percentage difference in the percentage of correct answers. In contrast, the group with the lowest percentage difference in correct answers was the retina, vitreous, and neuro-ophthalmology section. GPT-3.5 performance was significantly variable across the 13 subspecialties (p = 0.034). GPT-4.0 showed more consistent results across subspecialty groups than GPT-3.5 with no significant differences (p = 0.078). At the same time, GPT-3.5 had the highest percentage of correct answers in fundamentals (74%) and the lowest in pathology and tumors (53.0%). GPT-4.0 showed the highest percentage of correct answers in general medicine (88%) and the lowest rate of correct answers in clinical optics (70%). **Table 1** shows the amount and percentage of correct answers given by GPT-4.0 and GPT-3.5.

**Table 1:** Percentage and comparative statistical analysis of correct answers for GPT-4.0 and GPT-3.5 in different sub-specialties and areas of practice

Question Classification	Questions number (%)	GPT-4.0 correct answers (%)	GPT-3.5 correct answers (%)	GPT-4.0 vs. GPT- 3.5 P value
**All**	520 (100%)	408 (78.46% )	333 (64.15%)	0.0195
**Subspecialty**				
**Clinical optics**	40 (7.69%)	70%	55%	0.0156
**Cornea**	40 (7.69%)	78%	62%	0.0128
**Fundamentals**	40 (7.69%)	86%	74%	0.0265
**General medicine**	40 (7.69%)	88%	68%	< 0.01
**Glaucoma**	40 (7.69%)	73%	62%	0.0305
**Lens & Cataract**	40 (7.69%)	75%	65%	0.0336
**Neuro-Ophthalmology**	40 (7.69%)	75%	68%	0.0458
**Oculoplastic**	40 (%7.69)	72%	60%	0.0265
**Pathology & tumors**	40 (7.69%)	78%	53%	< 0.01
**Pediatrics**	40 (7.69%)	86%	68 %	< 0.01
**Refractive surgery**	40 (7.69%)	86%	66%	< 0.01
**Retina & vitreous**	40 (7.69%)	75%	68%	0.0458
**Uveitis**	40 (7.69%)	78%	65%	0.0238

## Discussion

This research provides promising new evidence of ChatGPT’s ability to handle complex clinical and medical data, particularly the development and consistency of artificial intelligence algorithms. AI chatbot technology has developed rapidly and is being used increasingly in e-society. ChatGPT, in particular, has become one of the fastest-growing computer applications in history, gaining 100 million active users in just 2 months [17].

Integrating AI into clinical practice and medical education has grown in popularity recently. Recent research indicates that the newest LLM versions exhibit a promising problem-solving capacity [18]. With its widespread use, it has been the subject of many studies, for example, one study reporting the relative success of ChatGPT on a sample United States Medical Licensing Examination (USMLE) Step 1 and Step 2 Clinical Knowledge assessment, achieving a passing threshold of approximately 60% [19]. The effectiveness of artificial intelligence was also studied in another board exam. In this study of the efficacy of artificial intelligence in the European Ophthalmology board exam, it was reported that GPT showed superior success by answering 6188 of 6785 questions correctly [16].

Very few studies in the literature show the performance of GPT-3.5 and GPT 4.0 against each other in ophthalmology [14,20]. In one of these studies, the GPT-4 was tested on two multiple-choice question sets of 260 questions from the Basic and Clinical Science Course (BCSC) Self-Assessment Program and OphthoQuestions question banks. The top-performing GPT-4 model was also contrasted with GPT-3.5 and past human performance. Antaki et al. found that GPT-4 significantly outperformed GPT-3.5 on simulated ophthalmology board-style exams, similar to the findings presented in this study [14]. In another study evaluating the ability to answer ophthalmology-related questions at different ophthalmology education levels, GPT-4.0 was found to perform significantly better than GPT-3.5 (75% vs 46%, p<0.01) [20].

In a relatively recent study, Moshirfar and colleagues evaluated human responses to 467 questions from GPT-4.0, GPT-3.5, and a question bank called StatPearls and obtained scores of GPT-4.0 73.2%, GPT-3.5 55.5%, humans 58.3%, respectively. Although it is not appropriate to directly compare this study and the presented study, Moshirfar et al. found that GPT-4.0 answered more questions correctly in percentage than GPT-3.5, similar to the results in this study [21].

This study found that GPT-4.0 answered more questions correctly than GPT-3.5, and the difference between the two groups was statistically significant (78.46% vs. 64.15%; p = 0.0195). Chat GPT 4.0 showed a statistically significant difference compared to Chat GPT 3.5 in giving correct answers in all subgroup analyses (p<0.05). In the subgroup analyses performed in this study, GPT-3.5 performance was significantly variable across the 13 subspecialties (p = 0.034). GPT-4.0 showed more consistent results across subspecialty groups than GPT-3.5 with no significant differences (p = 0.078). This result indicates that the GPT-4.0 algorithm is statistically more successful than GPT-3.5 in the ophthalmology question bank.

Finally, the statistically significant success of GPT-4.0 compared to GPT-3.5 in this study should be considered with the algorithm developments in the coming years, especially in online exams, which will increase gradually since the use of artificial intelligence is an increasing threat to test integrity. Thus, protocols such as mandatory proctoring should be considered.

### Limitation of Study

The first limitation of this study was that image—or video-based questions that could not be easily analyzed in ChatGPT-3.5, which was offered free of charge, were not evaluated. This limitation should be considered a limitation that might affect the study. Furthermore, the questions included in the study were not categorized as easy, medium, or complex. Even though the questions were chosen randomly, this factor should have also been considered statistically.

## Conclusion

The results of this study point to the potential for AI, and ChatGPT in particular, to positively contribute to medical education and practice. Moreover, the success of AI in its multiple-choice question bank exam could pave the way for greater integration of AI technology into medical education and continuing professional development. In the coming years, ChatGPT’s proficiency in clinical management and decision-making should be supported by further studies demonstrating that it can be a valuable resource for ophthalmologists and other medical professionals seeking information and guidance on complex cases. Furthermore, ChatGPT 4.0 was statistically more consistent and accurate in the study presented here than ChatGPT-3.5. AI technology, especially in ophthalmology, should be seen as a complement to, rather than a replacement for, medical professionals.

## Data sharing statement

All data produced in the present study are available upon reasonable request from the author. OphthoQuestions' question sets are proprietary and cannot be shared.
